# Efficacy of a structured auditory training module in older individuals with hearing loss: a randomized controlled trial

**DOI:** 10.1007/s00405-026-10058-6

**Published:** 2026-03-15

**Authors:** Archana Gundmi, Krishna Yerraguntla, Bellur Rajashekhar, Vasudeva Guddattu

**Affiliations:** 1 Department of Speech and Hearing, Manipal College of Health Professions, Manipal Academy of Higher Education, Manipal, 576104 Karnataka India; 2 Speech and Hearing Unit, King Khalid University, Abha, Saudi Arabia; 3 Department of Speech and Hearing, Manipal College of Health Professions, Manipal Academy of Higher Education (MAHE), Manipal, Karnataka India; 4 Department of Statistics, Manipal Academy of Higher Education, Manipal, Karnataka India

**Keywords:** Auditory training, Hearing loss, Older adults, Speech perception, Aural rehabilitation, Randomized controlled trial

## Abstract

**Purpose:**

To develop and evaluate a culturally adapted auditory training module for Kannada-speaking older adults with hearing loss.

**Methods:**

The initial phase focused on developing a structured Kannada auditory training module, followed by efficacy evaluation. Content was informed by the Listening and Communication Enhancement framework and refined through focused group discussions. Efficacy was assessed using a randomized controlled trial with a 2 × 2 factorial design. Eighty Kannada-speaking adults aged 45–65 years with bilateral mild-to-moderate sensorineural hearing loss were recruited. Participants were randomized using computer-generated permuted block allocation, stratified by hearing aid experience (< 1 year vs. >1 year), with blinded outcome assessment. The intervention group (*n* = 40) received 20 one-hour computer-based auditory training sessions over four weeks, while the control group (*n* = 40) underwent conventional aural rehabilitation. The primary outcome was improvement in speech perception under degraded listening conditions.

**Results:**

The intervention group showed significant improvements compared to controls. Quick Speech-in-Noise scores improved by 7–8 dB (F(1,76) = 142.10, *p* < 0.001), with 85% achieving clinically meaningful improvement (≥ 3 dB; NNT = 1.2). Compressed and filtered speech processing improved significantly (1.17–3.38-fold). Reaction time decreased for target word identification (4.95–5.45 s; F(1,76) = 93.46, *p* < 0.001) and missing word detection (F(1,76) = 77.88, *p* < 0.001). Self-reported hearing handicap reduced significantly (F(1,76) = 81.04, *p* < 0.001). No differences were observed between new and experienced hearing aid users.

**Conclusions:**

Structured auditory training significantly improves speech perception, cognitive processing speed, and self-reported outcomes in older adults with hearing loss, supporting its integration into aural rehabilitation for Kannada-speaking populations.

## Introduction

Aural rehabilitation aims to restore or enhance participation in activities limited by hearing impairment [1]. However, current practice for older adults with hearing loss predominantly emphasizes sensory management through hearing aids or cochlear implants, while other critical components instruction, training, and counselling receive insufficient attention [2]. A focused group discussion involving audiologists and hearing aid users in India revealed a strong consensus that hearing aid fitting alone is insufficient and that adults require structured auditory training to facilitate their communication skills [3]. Amplification devices improve audibility but cannot fully restore affected frequency resolution and temporal processing, resulting in auditory signals that remain inferior to normal hearing [4]. Consequently, individuals with hearing loss require higher signal-to-noise ratios (SNR) than normal-hearing individuals [5], significantly limiting hearing aid benefit.

Recent research has increasingly recognized cognitive ability as a key predictor of hearing aid benefit [6, 7]. Listening in noise is a complex task requiring selective attention to target speech while ignoring competing signals and demands substantial cognitive resources. These cognitive processing skills, essential for speech perception in degraded acoustic conditions, often deteriorate with age-related hearing loss [7]. This cognitive-auditory interaction underscores the need for rehabilitation approaches that address both auditory and cognitive domains [8]. Recent advances in auditory rehabilitation have increasingly emphasized the interconnected nature of hearing and cognitive health, with comprehensive approaches demonstrating improvements in quality of life for older adults beyond basic amplification [9, 10].

The effectiveness of auditory training programs has generated considerable debate, with studies showing mixed results. While some investigations demonstrated positive outcomes [11, 12], others reported limited effects or poor generalization [13–16]. Despite these limitations, emerging evidence suggests that appropriately designed auditory training can induce neuroplastic changes improving speech perception beyond trained stimuli [17]. Anderson and Kraus [17] demonstrated that auditory training in older adults produces measurable changes in neural encoding of speech sounds, particularly in challenging listening environments, supporting the biological plausibility of training-induced benefits. Recent systematic reviews and meta-analyses have identified key success factors including training intensity, individual cognitive capacity, and stimulus variability [18–21].

Contemporary neuroscience research has further elucidated the mechanisms underlying training benefits. Neuroimaging studies reveal that auditory training strengthens neural networks involved in both auditory processing and cognitive control [22, 23], explaining the dual benefits observed in speech perception and cognitive function. Recent work by Bidelman and Yoo [24] demonstrated enhanced subcortical speech encoding following auditory training in older adults, underscoring the importance of training programs that address both peripheral and central auditory processing mechanisms.

Technology integration has enhanced auditory rehabilitation accessibility and effectiveness. Computer-based programs offer structured, portable, and data-driven therapeutic approaches, with recent developments including advanced platforms and mobile health applications [25–27]. Teleaudiology services have emerged as viable delivery models for hearing aid rehabilitation, expanding access particularly in underserved populations [28]. Mobile-based auditory training applications have been developed and validated for older adults with hearing impairments, demonstrating user-friendliness and compatibility with the target population [29]. However, in India, software development has focused predominantly on speech-language pathology, with limited attention to auditory rehabilitation for adults. Structured auditory training programs for Kannada-speaking older adults remain underdeveloped. This gap is particularly concerning given India’s aging population and high prevalence of hearing impairment—66.9% among elderly populations in Karnataka state [30, 31].

The growing need for adult auditory rehabilitation in India reflects increased longevity and hearing aid adoption among older adults seeking better quality of life. However, optimized hearing aid fitting alone rarely achieves user satisfaction, necessitating structured training programs as standard practice [32, 33]. This study developed and evaluated a culturally adapted auditory training module for Kannada-speaking older adults with hearing loss, addressing the significant gap in evidence-based aural rehabilitation resources for Indian populations.

## Methods

### Study design and ethics approval

**Fig. 1:**
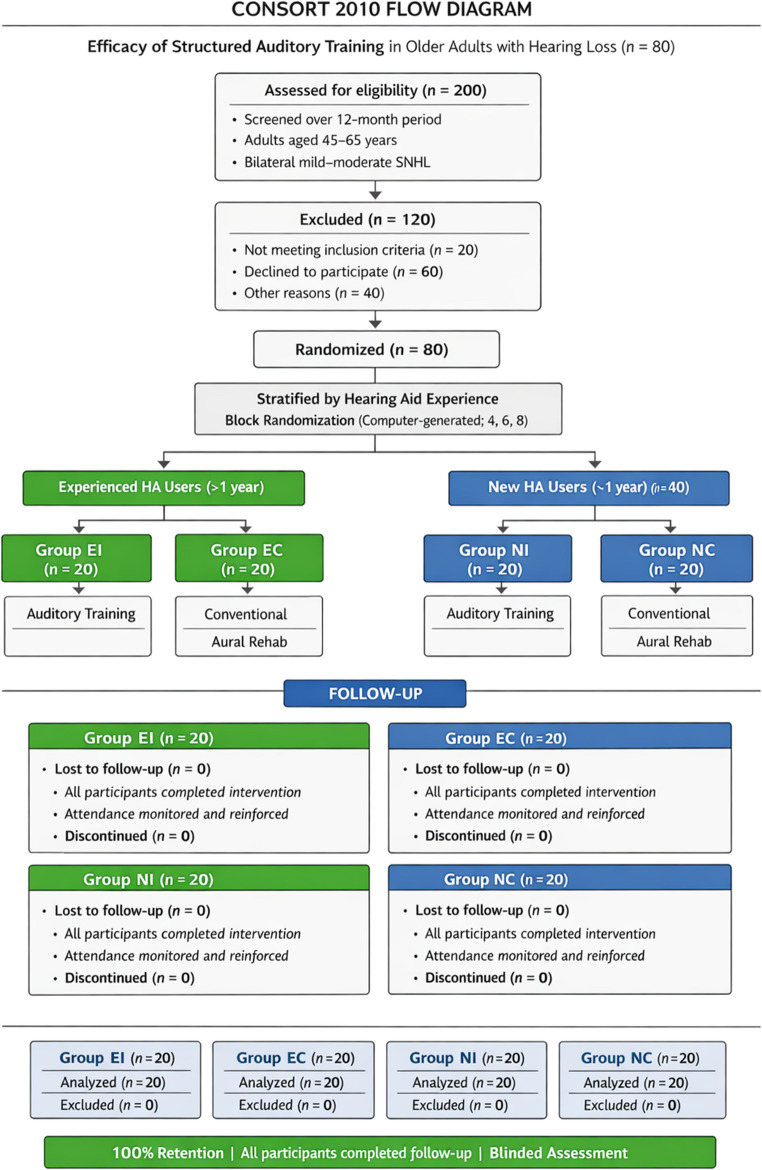
CONSORT 2010 flow diagram illustrating participant recruitment, eligibility assessment, stratified randomization based on hearing aid experience, allocation to auditory training and conventional aural rehabilitation groups, follow-up, and final analysis

As the first phase of the study, the Kannada auditory training module was adapted from the Listening And Communication Enhancement (LACE) program with permission from the original authors. The adaptation process addressed the linguistic, cultural, and demographic characteristics of Kannada speakers in Karnataka, India. Content validity was established through a rigorous three-round expert review involving a focus group of five audiologists (more than 10 years experience), three Dravidian linguistics experts, and two cognitive psychologists. Feedback was quantified using the Content Validity Ratio (CVR) and Content Validity Index (CVI). Only items reaching high statistical consensus regarding essentiality and relevance were included in the final software-based module, which covered three domains: Degraded Speech, Cognitive Skills, and Communication Strategies. In the second phase, the study employed a randomized controlled trial design with a 2 × 2 factorial structure, examining the effects of auditory training across two participant groups (new vs. experienced hearing aid users) and two intervention conditions (training vs. control). Ethics approval was obtained (Protocol No: IEC 210/2013), and the study was conducted in accordance with the Declaration of Helsinki principles for human research. The CONSORT flow diagram is presented in Fig. 1.

### Sample size calculation and participant recruitment

Sample size calculation was performed using G*Power 3.1.9.2 software based on pilot data and previous literature. With an expected effect size of d = 0.8 for the primary outcome measure (Quick SIN-K), alpha level of 0.05, and power of 80%, a minimum sample size of 17 participants per group was required. To account for potential dropout and ensure adequate power for subgroup analyses, 20 participants per group were recruited, resulting in a total sample of 80 participants. Participants were recruited through systematic screening of patients attending an audiology clinic at a medical college hospital over a 12-month period.

Inclusion criteria:

- Age between 45 and 65 years.
- Bilateral sensorineural hearing loss of mild to moderate degree (pure tone average 26–70 dB HL for 500, 1000, 2000, and 4000 Hz).
- Native Kannada speakers with Kannada as primary language of communication.
- Current hearing aid users for either less than one year (new users) or more than one year (experienced users).
- Cognitive capacity sufficient to complete training tasks as assessed by Mini-Mental State Examination score ≥ 24.

### Randomization and group allocation

The randomization process employed computer-generated random sequence allocation using permuted blocks of varying sizes (4, 6, 8) to ensure balanced group assignment while maintaining allocation concealment. Randomization was stratified by hearing aid experience (new vs. experienced) to ensure equal distribution across intervention groups. Sealed opaque envelopes containing group allocations were prepared by an independent researcher not involved in participant recruitment or assessment.

### Baseline evaluation

A set of standardized tests was administered after the routine audiological evaluation for all participants. The battery of tests included: Quick Speech-in-Noise test in Kannada (Quick-SIN-K), Low pass filter speech test in Kannada (LFT-K), Compressed Speech test in Kannada (CST-K), Hearing Handicap Inventory for Adults in Kannada (HHIA-K), and reaction time calculated for both working memory and speed of processing.

All tests except HHIA-K were presented through the calibrated audiometer with the participant seated at a distance of three feet from the loudspeaker at zero degree azimuth in the free field at most comfortable level. Along with these tests, the average reaction time before and after training was noted for both missing word task and target word task under the cognitive task domain as outcome measures. All tests were administered in the same order for all participants with 30 min gap between tests.

### Intervention protocol

#### Training group (intervention)

Following baseline evaluation, participants were divided into control and intervention groups based on randomization, in both new and experienced hearing aid users. The control group was enrolled for traditional aural rehabilitation, which included fitting a hearing aid followed by counseling on different communication strategies. Listening training was given to the intervention group using the developed software by an experienced audiologist. Auditory training was given using a laptop (DELL Inspiron 1440, USA). During training, stimulus was presented through a loudspeaker connected to the laptop via sound cards and measured using SLM (Linear network) at zero degree azimuth at a distance of three feet from the loudspeaker. The desired intensity level was set by the participant as their comfortable level. Throughout the session, the same loudness level was maintained. Therapy was taken in a quiet room with noise level less than 40 dB SPL.

The participants in the intervention group underwent therapy of five sessions each week of one-hour duration. During the session, training was focused majorly on degraded speech followed by cognitive task and compensatory strategies. Feedback was provided in every session depending on the errors made by them. All sessions were concluded by discussing their progress and goals for the next session. All participants were given a total of 20 sessions of therapy. A minimum of 75% of the task in the module was completed by all participants in the intervention group.

Each one-hour session was structured to transition from counseling to intensive task-based training. The first 10 min focused on a clinician-patient dialogue regarding daily conversational experiences to establish rapport and monitor real-world progress. This was followed by 40 min of targeted auditory exercises, primarily emphasizing speech perception in noise and degraded listening conditions with adaptive difficulty. The final 10 min were dedicated to a structured review where the clinician and patient discussed specific communication difficulties encountered during the week, applying learned repair strategies to solve these personal challenges.

### Blinding procedures

Outcome assessors were blinded to group allocation throughout the study. All outcome measures were administered by audiologists who were not involved in delivering the interventions and were unaware of participant group assignments. Participants were instructed not to discuss their intervention experiences with assessors. The success of blinding was not formally assessed.

### Statistical analysis

Descriptive statistics (mean and standard deviation) summarized all outcome measures. Distribution normality was assessed using Shapiro–Wilk tests and visual inspection of Q–Q plots. Quick SIN-K, reaction time measures, and HHIA-K scores followed normal distributions and were analyzed using repeated measures Analysis of Variance (ANOVA) with time (pre/post) as within-subjects factor and group (intervention/control) and hearing aid experience (new/experienced) as between-subjects factors. Within-group mean differences with 95% confidence intervals served as effect size measures.

Compressed speech and filtered speech scores did not follow normal distributions and were analyzed using Poisson regression models with robust standard errors. Effect sizes were calculated using Cohen’s d for parametric measures and Cliff’s delta for non-parametric measures.

Minimal Clinically Important Difference (MCID) analysis determined the proportion of participants achieving clinically meaningful improvements (≥ 3 dB for Quick-SIN-K). Number Needed to Treat (NNT) with 95% confidence intervals was calculated for primary outcomes. All analyses were performed using SPSS version 22.0 with alpha level set at 0.05.

## Results

### Participant characteristics and baseline comparability

Eighty participants successfully completed the study, achieving a 100% retention rate. The mean age of participants ranged from 50.7 to 55.6 years across the different groups. The gender distribution was relatively balanced, comprising 46 males and 34 females in total. All participants exhibited bilateral mild to moderate-severe sensorineural hearing loss. For new users, hearing aid usage varied from 6 months to 1 year, while experienced users averaged between 4.9 and 8.5 years of use. There was a range of hearing aid fitting patterns, with both unilateral and bilateral configurations represented across groups. Importantly, there were no significant baseline differences in age, hearing thresholds, or initial outcome measure scores among the groups, confirming successful randomization.

### Primary outcome: speech-in-noise performance

Quick Speech-in-Noise test in Kannada (Quick-SIN-K) scores are presented in Table 1. Initial comparison between experienced hearing aid users (Group E) and new hearing aid users (Group N) revealed no statistically significant differences in training effects (F(1,76) = 0.517, *p* = 0.474). Therefore, these groups were combined for subsequent analysis comparing intervention and control conditions.

**Table 1:** Mean and standard deviation of Quick SIN-K scores from baseline to follow-up across different groups

Type of hearing aid usage	Groups	Pre Quick SIN-K dB SNR Mean ± SD	Post Quick SIN-K dB SNR Mean ± SD	Difference in dB SNR 95% CI	F(df1,df2), *p* value*
Experienced Hearing aid users (Group E)	Intervention (Group EI)	19.27 ± 4.58	10.52 ± 2.53	8.75 (6.38, 11.11)	F(1,76) = 142.10, *p* < 0.001
	Control (Group EC)	18.12 ± 3.72	17.72 ± 3.66	0.4 (− 1.72, 2.82)	
New Hearing aid users (Group N)	Intervention (Group NI)	16.72 ± 4.8	8.32 ± 1.72	8.4 (6.09, 10.71)	
	Control (Group NC)	19.27 ± 3.85	18.27 ± 3.42	1.0 (− 1.33, 3.33)	

*p value for comparison of intervention and control groups

The intervention group demonstrated significant improvement in Quick-SIN-K scores compared to the control group (F(1,76) = 142.10, *p* < 0.001). For experienced hearing aid users, the intervention group improved by a mean of 8.75 dB SNR (95% CI: 6.38–11.11), with scores decreasing from 19.27 ± 4.58 dB SNR at baseline to 10.52 ± 2.53 dB SNR at follow-up. In contrast, the control group showed minimal change (mean difference: 0.4 dB, 95% CI: −1.72 to 2.82). Similarly, for new hearing aid users, the intervention group improved by 8.4 dB SNR (95% CI: 6.09–10.71), with scores decreasing from 16.72 ± 4.8 dB SNR to 8.32 ± 1.72 dB SNR, while the control group showed only 1.0 dB improvement (95% CI: −1.33 to 3.33).

Minimal Clinically Important Difference (MCID) analysis revealed that 85% of intervention participants achieved clinically meaningful improvement (≥ 3 dB), compared to only 15% in the control group. The Number Needed to Treat (NNT) was 1.2 (95% CI: 1.1–1.4), indicating that approximately one to two participants needed to receive the intervention for one additional participant to achieve clinically meaningful benefit.

### Secondary outcomes: speech processing measures

#### Compressed speech performance

Compressed Speech Test-Kannada (CST-K) scores demonstrated significant improvements in the intervention group across all compression ratios. For experienced hearing aid users in the intervention group (Group EI), the mean score at 50% compression increased from 62.10 at baseline to 69.90 at follow-up, representing a 1.13-fold improvement. The mean ratio of improvement from baseline to follow-up in Group EI was 1.17 times higher than the control group (Group EC) at 50% compression. At higher compression levels, the advantage of training became more pronounced, with Group EI showing 1.27, 1.35, and 3.38 times higher improvement ratios than Group EC at 60%, 70%, and 80% compression, respectively.

For new hearing aid users in the intervention group (Group NI), the mean compression score at 50% showed a 1.23-fold improvement from baseline to follow-up. The mean ratio was 1.26 times higher in the intervention group compared to the control group at 50% compression. Similar patterns of greater improvement in the intervention group were observed at 60%, 70%, and 80% compression levels.

Wald chi-square tests comparing experienced versus new hearing aid users revealed no significant differences between these groups (χ²(3) = 1.27, *p* = 0.734), indicating that training benefits were comparable regardless of hearing aid experience. Poisson regression analysis confirmed statistically significant effects of training on compressed speech performance across all compression ratios.

#### Low-pass filtered speech performance

Low-pass Filtered Speech Test-Kannada (LFT-K) results showed significant improvements in the intervention group. For experienced hearing aid users, the intervention group (Group EI) demonstrated a 1.19-fold improvement at 2500 Hz from baseline to follow-up, compared to 0.96-fold change in the control group (Group EC). The mean ratio at 2500 Hz was 1.27 times higher in the intervention group compared to controls. Similar patterns were observed at other filter frequencies (2000 Hz, 1500 Hz, and 800 Hz), with progressively greater training effects at lower cut-off frequencies.

For new hearing aid users, the intervention group (Group NI) showed a 1.38-fold improvement at 2500 Hz from baseline to follow-up evaluation. Improvements were observed across all filter frequencies, with the magnitude of improvement increasing as the cut-off frequency decreased, reflecting enhanced ability to process degraded spectral information.

Wald chi-square tests revealed no significant differences between experienced and new hearing aid users (χ²(3) = 0.326, *p* = 0.95), confirming that training benefits generalized across hearing aid experience levels. Poisson regression analysis demonstrated statistically significant training effects on filtered speech perception.

### Cognitive performance measures

#### Reaction time for target (RTT) word identification

Both experienced and new hearing aid users in the intervention group showed substantial reductions in reaction time compared to controls. For experienced users, the intervention group reduced RTT by a mean of 4.95 s (95% CI: 2.78–7.13), from 10.80 ± 3.96 s at baseline to 5.85 ± 2.73 s at follow-up. The control group showed minimal change (mean difference: 0.23 s, 95% CI: 1.96–2.41).

For new hearing aid users, the intervention group demonstrated a mean reduction of 5.45 s in RTT (95% CI: 4.17–8.12), decreasing from 11.60 ± 3.03 s to 5.45 ± 3.15 s. The control group again showed minimal improvement (mean difference: 0.35 s, 95% CI: 1.26–1.96).

Repeated measures ANOVA revealed no significant differences between experienced and new hearing aid users (F(1,76) = 0.967, *p* = 0.329), but highly significant differences between intervention and control groups (F(1,76) = 93.46, *p* < 0.001), indicating substantial training effects on processing speed regardless of hearing aid experience.

#### Reaction time for missing word detection

Reaction time for missing word (RTM) detection followed similar patterns to target word identification. The intervention group demonstrated significantly faster reaction times at follow-up compared to controls in both experienced and new hearing aid user groups. Statistical analysis using repeated measures ANOVA showed no significant differences between hearing aid experience groups (F(1,76) = 0.227, *p* = 0.635), but highly significant differences between intervention and control groups (F(1,76) = 77.88, *p* < 0.001).

These findings indicate that auditory training enhanced cognitive processing speed for both target identification and missing word detection tasks, with benefits generalizing across hearing aid experience levels.

### Self-reported hearing handicap

Hearing Handicap Inventory for Adults-Kannada (HHIA-K) scores decreased significantly in the intervention group compared to controls, indicating reduced self-perceived hearing handicap. Both experienced and new hearing aid users in the intervention group reported substantial reductions in hearing-related difficulties across emotional and situational domains.

Repeated measures ANOVA revealed no significant differences in HHIA-K score changes between experienced and new hearing aid users (F(1,76) = 0.341, *p* = 0.561). However, comparison between intervention and control groups showed highly significant differences (F(1,76) = 81.04, *p* < 0.001), with the intervention group reporting greater reductions in hearing handicap than controls. These findings demonstrate that structured auditory training significantly improved participants’ quality of life and reduced the psychosocial impact of hearing loss, independent of hearing aid experience.

## Discussion

### Primary findings and clinical significance

This randomized controlled trial provides robust evidence that structured, intensive auditory training produces clinically and statistically significant improvements in speech perception in noise, rapid and filtered speech processing, cognitive processing speed, and self-reported hearing-related quality of life in older adults with hearing loss. Participants in the intervention group demonstrated a mean improvement of 7–8 dB in signal-to-noise ratio (SNR) on the QuickSIN-K test, with 85% achieving clinically meaningful improvement (≥ 3 dB) and a remarkably low Number Needed to Treat (NNT = 1.2). These findings strongly support the clinical utility and efficiency of structured auditory training as a core component of comprehensive aural rehabilitation.

The magnitude of improvement observed exceeds that reported in much of the previous literature, where gains in speech-in-noise performance typically range between 1 and 3 dB [13, 34, 35]. Given estimates that each 1 dB improvement in SNR corresponds to a 6–8% increase in speech identification [36, 37], the observed improvement in the present study could translate to an approximate 40% functional gain in everyday communication. Such gains are likely to be perceptually meaningful and impactful in real-world listening environments, addressing the fundamental limitation that individuals with hearing loss require higher signal-to-noise ratios than normal-hearing individuals even with optimal amplification.

### Reconciling mixed evidence in the auditory training literature

The present findings contribute important clarity to the ongoing debate regarding the efficacy of auditory training in adults with hearing loss. While earlier studies have reported limited effects or poor generalization [13–16], recent systematic reviews and meta-analyses increasingly support the effectiveness of auditory and cognitive training when appropriately designed [18–21, 38].

Several methodological factors may explain discrepancies between the present findings and earlier negative reports. First, training intensity and dosage appear critical. The present protocol involved 20 one-hour sessions delivered over four weeks, a substantially higher training dose than many previous investigations. A recent meta-analysis by Lawrence et al. [18] demonstrated a clear dose–response relationship, with higher training intensity predicting larger effect sizes in both auditory and cognitive outcomes. This suggests that insufficient training exposure may have limited neuroplastic changes in earlier studies that reported null or modest findings. Recent reviews have further emphasized methodological considerations for auditory training interventions, highlighting the importance of rigorous study design, appropriate control conditions, and ecologically valid outcome measures [20].

Second, the comprehensive nature of the training program may have contributed to the observed benefits. Unlike single-domain approaches focusing exclusively on speech-in-noise perception, the present intervention simultaneously targeted degraded speech perception, rapid speech processing, filtered speech, cognitive speed, and communication strategies. This multidomain approach addresses both peripheral and central auditory processing mechanisms. Multidomain training has been shown to produce synergistic benefits by engaging overlapping auditory cognitive networks [17, 18, 38], thereby enhancing generalization to real-world listening situations a critical limitation identified in earlier single-domain studies.

Third, linguistic and cultural adaptation likely enhanced engagement and transfer of learning. Training materials were developed in Kannada, incorporated culturally relevant communication contexts, and were delivered using native speech stimuli. Ecological validity and cultural relevance are increasingly recognized as critical determinants of rehabilitation success [39, 40]. Studies relying on English-language materials in non-native populations may inadvertently underestimate training benefits due to reduced relevance and engagement, particularly in diverse linguistic contexts like India.

Fourth, participant characteristics differed notably from previous studies. The current study included individuals with greater severity of hearing loss and a younger age ceiling (65 years versus 85 years). The higher baseline impairment may have provided greater opportunity for improvement, while the younger age range likely minimized ceiling effects that could have constrained performance gains in older cohorts.

### Neuroplastic mechanisms underlying training benefits

The broad improvements observed across perceptual, cognitive, and self-reported outcomes support the hypothesis that intensive auditory training induces experience-dependent neuroplastic changes within the auditory and cognitive systems. These findings align with contemporary neuroscience research demonstrating that auditory training can produce measurable changes in neural encoding of speech sounds, particularly in challenging listening environments [17, 24, 25].

Improvements in reaction time measures for both target word identification and missing word detection suggest enhanced processing efficiency and working memory. Recent neuroimaging and electrophysiological evidence indicates that auditory training strengthens neural networks involved in both auditory processing and cognitive control [23, 25], explaining the dual benefits observed in speech perception and cognitive function. These findings provide a mechanistic explanation for the observed transfer of training benefits beyond auditory perception alone. Studies have demonstrated that structured training can reduce the cognitive load associated with listening in challenging environments and modify not only perceptual abilities but also listening comfort and tolerance [21, 41].

Importantly, the absence of differential training effects between new and experienced hearing aid users suggests that auditory neuroplasticity remains accessible even beyond the initial acclimatization period. This finding aligns with contemporary models of lifelong plasticity, which emphasize that older adults retain the capacity for functional reorganization with appropriate stimulation [42, 43].

### Implications for comprehensive aural rehabilitation and technology integration

The present findings have significant implications for clinical practice. Amplification devices improve audibility but cannot fully restore frequency resolution and temporal processing. Structured auditory training should therefore be viewed as an essential component of evidence-based aural rehabilitation alongside instruction and counselling [1, 2]. The exceptionally low NNT (1.2) highlights the efficiency and cost-effectiveness of implementing such programs. Recent systematic reviews have confirmed that multifaceted interventions incorporating amplification, training, and counseling produce superior outcomes compared to device fitting alone [9].

The finding that training benefits are independent of hearing aid experience provides clinicians with flexibility in timing intervention delivery, accommodating patient readiness and clinical logistics. This flexibility is enhanced through technology-mediated delivery platforms. The success of the computer-based training module demonstrates the feasibility of digital rehabilitation, with recent studies showing promising outcomes using mobile and web-based applications [27, 29, 33, 39]. Teleaudiology services have demonstrated effectiveness for hearing aid fitting and follow-up care in adult populations, though considerations regarding technology access and clinical appropriateness remain important [28]. These platforms offer standardized stimulus delivery, adaptive progression, objective tracking, and scalability through home-based models.

### Cultural and linguistic relevance

The superior outcomes observed in this study underscore the importance of culturally and linguistically tailored rehabilitation interventions. The systematic three-round expert validation process, incorporating audiologists, Dravidian linguistics experts, and cognitive psychologists, ensured that training materials reflected authentic communicative contexts and linguistic features specific to Kannada speakers. This ecological validity likely enhanced participant engagement, motivation, and transfer of learning to real-world situations—factors that may partially explain the larger effect sizes compared to previous studies using standardized English-language protocols in diverse populations.

The Kannada adaptation extended beyond simple translation to include culturally relevant communication scenarios, appropriate social contexts, and dialectal variations common in Karnataka. This cultural resonance may have contributed to the 100% retention rate and high adherence (minimum 75% task completion) observed in the intervention group. These findings have important implications for rehabilitation in multilingual contexts. The methodological framework employed in this study—expert validation, focused group input, and rigorous content validity assessment—provides a replicable template for developing auditory training programs in other Indian languages and low-resource settings globally. Promoting hearing and cognitive health in audiologic rehabilitation requires integration of evidence-based practices with culturally sensitive implementation that acknowledges diverse healthcare beliefs, family structures, and communication styles [10]. Investment in linguistically diverse rehabilitation resources represents both a clinical imperative and an opportunity to advance equitable access to evidence-based interventions across heterogeneous populations.

### Limitations and future directions

Several limitations should be acknowledged. Long-term maintenance of training benefits beyond the four-week follow-up was not assessed. Additionally, although outcome assessors were blinded, participant blinding was not feasible. Generalizability to individuals with more severe hearing loss, cognitive impairment, or different linguistic backgrounds warrants further investigation.

Future research should explore long-term retention of benefits, optimal training dosage, personalization based on auditory–cognitive profiles and individual cognitive capacity, cost-effectiveness analyses, and validation of similar programs across diverse linguistic populations. Additionally, investigation of hybrid delivery models and mobile health applications could further enhance accessibility and implementation in resource-limited settings [43, 44].

## Conclusion

This study provides strong evidence that structured, intensive auditory training produces clinically meaningful improvements across perceptual, cognitive, and functional domains in older adults with hearing loss. The magnitude of benefit observed, coupled with the low NNT and applicability across different hearing aid user types, supports the integration of auditory training as a core component of comprehensive aural rehabilitation. Technology-enabled, culturally adapted training programs offer a scalable approach to addressing the substantial burden of hearing-related communication difficulties in aging populations.
